# Early pembrolizumab clearance as prognostic biomarker for non‐response in patients with advanced non‐small cell lung cancer

**DOI:** 10.1002/ijc.70052

**Published:** 2025-07-24

**Authors:** Fenna de Vries, Leila‐Sophie Otten, Berber Piet, Eric J. F. Franssen, Arthur A. J. Smit, Michel M. van den Heuvel, Rob ter Heine, The DEDICATION‐1 Study Group, Marijn Smits‐Zwinkels, Erica Geraedts, Jeske Staal‐van den Brekel, Yvonne Berk, Femke van der Meer, Niels Claessens, Bianca van Veggel, Lizza Hendriks, Wouter Jacobs, Annette Bijsmans, Arthur Mulders, Cor van der Leest, Joost Jansen, Wouter van Geffen, Quincy de Waard, Maggy Youssef, Wai Yee Lam‐Wong, Keetie van Loenhout, Daphne Dumoulin, Suzi Samii, Svitlana Tarasevych, Christi Steendam, Vivian van Kampen‐van den Boogaart, Judith Herder, Ernst Lammers, T. Jeroen N. Hiltermann, Nicole Barlo, Emanual Citgez, Antoinette Kroeze

**Affiliations:** ^1^ Department of Pharmacy OLVG Amsterdam North‐Holland The Netherlands; ^2^ Department of Pharmacy Research Institute for Medical Innovation, Radboudumc Nijmegen Gelderland the Netherlands; ^3^ Department of Respiratory Medicine Research Institute for Medical Innovation, Radboudumc Nijmegen Gelderland the Netherlands; ^4^ Department of Pulmonology OLVG Amsterdam North‐Holland The Netherlands; ^5^ Department of Pulmonology University Medical Center Utrecht Utrecht the Netherlands

**Keywords:** biomarker, immune checkpoint inhibitors, lung cancer

## Abstract

Immune checkpoint inhibitors have improved survival rates in patients with advanced‐stage non‐small cell lung cancer; however, the majority obtain no long‐term benefits. We investigated pembrolizumab clearance as an early prognostic biomarker and evaluated its accuracy using a limited sampling strategy. Pembrolizumab clearance was calculated using non‐linear mixed effects modeling, and cut points were determined using maximally selected rank statistics. The prognostic value for survival was estimated using univariate Cox regression analysis. Sensitivity, specificity, and positive and negative predictive values were calculated to evaluate the performance in identifying response (defined as disease control at 6 months). The accuracy of a limited sampling strategy was evaluated using MPE and NRSME. Among 303 patients included, 65% experienced disease progression, and 60% died. Patients with pembrolizumab clearance above 0.232 L/day at the first dose were more likely to have disease progression (HR = 1.98, 95% CI [1.21, 3.26], *p* = .007) or poor survival (HR = 2.04, 95% CI [1.16, 3.59], *p* = .014). A diminished decrease in clearance (<15.8%) at 6 weeks was also significantly associated with progression (HR = 1.46, 95% CI [1.12, 1.92], *p* = .006) and poor survival (HR = 1.82, 95% CI [1.35, 2.45], *p* = .000). Pembrolizumab clearance showed high sensitivity (0.96, 95% CI [0.92, 0.99]), but moderate positive predictive value (0.48). Limited sampling matched comprehensive sampling accuracy (MPE = +4.5% vs. +3.2%, NRSME = 16.8% vs. 14.2%). Early pembrolizumab clearance is a feasible prognostic biomarker for survival, with opportunities to enhance its positive predictive value before clinical implementation.

## What's New?

Immune checkpoint inhibitors have improved survival rates in patients with advanced‐stage non‐small cell lung cancer; however, the majority obtain no long‐term benefits. This analysis derived from two prospective clinical studies indicates that lean body weight‐normalized clearance of pembrolizumab is a highly sensitive biomarker for the identification of non‐responders to immune checkpoint inhibitors. Requiring only two samples during early treatment for precise assessment, pembrolizumab clearance emerges as a clinically practical prognostic biomarker for survival. The new approach could potentially help stratify patients at an early treatment stage and timely inform therapeutic decisions in non‐small cell lung cancer.

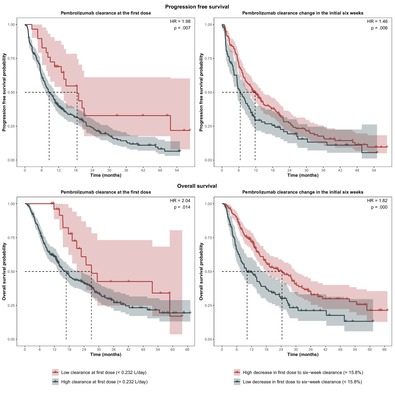

**Abbreviations**
ECOG PS: Eastern Cooperative Oncology Group Performance Status
ELISA: Enzyme‐linked immunosorbent assay
ICI: Immune checkpoint inhibitor
NPV: Negative predictive value
NSCLC: Non‐small cell lung cancer
OS: Overall survival
PD‐1: Programmed cell death protein 1
PD‐L1: Programmed cell death ligand 1
PFS: Progression‐free survival
PPV: Positive predictive value
RECIST 1.1: Response Evaluation Criteria in Solid Tumors 1.1

## 1 BACKGROUND

Lung cancer remains the most commonly diagnosed malignancy worldwide, with nearly 2.5 million new cases reported in 2022. It is also the leading cause of cancer‐related deaths, accounting for approximately 1.8 million fatalities annually.^1^ Among the various types of lung cancer, non‐small cell lung cancer (NSCLC) is the most prevalent, comprising nearly three‐quarters of all lung cancer cases globally in 2020.^2^ Historically, overall survival (OS) was poor, with median OS ranging between 8 and 14 months.^3^, ^4^ New therapies such as immune checkpoint inhibitors (ICI) significantly improved survival, but only a minority of patients benefit long term.^5^

ICIs such as pembrolizumab, a monoclonal antibody that targets the programmed cell death‐1 (PD‐1) receptor, have emerged as the cornerstone in treating advanced NSCLC. Since its introduction in 2014, its use has expanded significantly.^6^, ^7^, ^8^ Despite the widespread adoption, the overall response rate for ICIs targeting PD‐1 or its ligand (PD‐L1) remains modest, at around 37.5% in first‐line treatment.^5^ Given these limited response rates, early biomarkers are urgently needed to identify non‐response and guide treatment decisions. Given the often short window of opportunity in this rapidly progressing disease, an early switch to an alternative treatment or cessation of an ineffective treatment may increase the efficacy of treatment and reduce unnecessary exposition to an ineffective drug.

Recent studies suggest that antibody clearance may be a valuable prognostic biomarker.^9^, ^10^ Higher pembrolizumab clearance at the first dose has been associated with poorer OS, and a decrease in pembrolizumab clearance observed during treatment correlates with the response to therapy.^11^, ^12^ These dynamic changes in clearance result from broader physiological changes associated with cancer progression rather than from pharmacokinetic interactions between drug exposure and outcomes.^11^, ^13^ Cancer cachexia and cancer‐associated inflammation are thought to lead to increased catabolic clearance of proteins, including therapeutic antibodies. This hypercatabolic state has been associated with treatment failure and poor survival outcomes.^14^, ^15^

Currently, there is a lack of data supporting pembrolizumab clearance as a prognostic biomarker in clinical practice. We, therefore, aimed to assess early pembrolizumab clearance as a prognostic biomarker for survival in patients with NSCLC, whereby we hypothesize that higher early pembrolizumab clearance and a diminished decline in clearance over time are associated with poorer survival outcomes and treatment non‐response.

## 2 METHODS

### 2.1 Study population and data collection

The study population is derived from two prospective clinical studies: the DEDICATION‐1 study and the PD‐1 study. Patients in the DEDICATION‐1 study were enrolled between December 2020 and October 2022 in Radboud University Medical Center and other participating institutions in the Netherlands, while patients in the PD‐1 study were enrolled between October 2020 and September 2022 in OLVG. In both studies, inclusion was consecutively and aligned with routine clinical care.^16^ There were some additional exclusion criteria for the DEDICATION‐1 study, with the major exclusion criteria being prior ICI treatment, chemotherapy within 3 months before enrollment, history of solid organ transplantation, and body weight below 40 kg or above 140 kg. Additionally, for inclusion in this current analysis, all patients should have at least one quantified pembrolizumab plasma concentration above the assay's lower limit of quantification and no uncertainties regarding the sampling moment.

Baseline data collection included gender, age, height, weight, estimated glomerular filtration rate, albumin level, smoking history (current, former, never smoker, and pack‐years), and PD‐L1 expression (0–49% or ≥50%) and Eastern Cooperative Oncology Group Performance Status (ECOG PS). During the study, data on treatment administration time and date, treatment regimen (pembrolizumab monotherapy, pembrolizumab‐pemetrexed‐platinum, or pembrolizumab‐paclitaxel‐platinum), pembrolizumab dose, sampling date and time, body weight (within 2 weeks of infusion or blood sampling), and ECOG PS were collected. Furthermore, survival data were derived from routine clinical care. Response evaluation was performed according to Response Evaluation Criteria in Solid Tumors (RECIST) 1.1 criteria, as per standard clinical practice.^1^, ^17^

Patients were censored for progression‐free survival (PFS) if they were alive at the data analysis cut‐off date (June 2024), were lost to follow‐up or died from unknown causes, and showed no disease progression. Overall survival was defined as the time from the first pembrolizumab dose to death from any cause. Patients lost to follow‐up or alive at the data cut‐off were censored for OS analysis. Follow‐up duration was specified as the time from the first pembrolizumab dose to when patients were lost to follow‐up, death, or the data cut‐off. Pharmacokinetic samples were obtained consisting of peak (directly after infusion), mid (halfway of each 6‐week cycle), trough (just before administration), and post‐treatment samples (up to hundred days after discontinuation). Further details for the bioanalysis are presented in Data S1 (Materials and Methods S1) and Figure S1.

### 2.2 Early clearance as a prognostic biomarker for survival

#### 2.2.1 Pharmacokinetic modeling of pembrolizumab clearance

Individual empirical Bayes estimates for pembrolizumab clearance were obtained using non‐linear mixed effects modeling. In short, a two‐compartment pharmacokinetic model, accounting for the known time‐varying clearance of pembrolizumab, was fitted to the data, as described in detail in Data S1 (Materials and Methods S2), Table S1 and Figures S2.1, S2.2, and S2.3. All other statistical analyses were performed using R version 4.1.3, with Rstudio version 2022.02.1 as the interface, and using packages “survminer”, “survival”, “ggplot2”, and “maxstat”.^18^, ^19^, ^20^, ^21^

The individual empirical Bayes estimates for pembrolizumab clearance were normalized to a lean body weight of 52.5 kg to account for the effect of weight on clearance and allow comparison of pembrolizumab clearance for individuals with different body sizes. As pembrolizumab clearance at the first dose reflects the patient's condition at the start of treatment,^12^, ^22^ estimation of lean body weight normalized clearance at this time point was chosen to assess the prognostic value of pembrolizumab clearance for survival. In addition, as the extent of early change of pembrolizumab clearance might be indicative of the primary response to treatment^13^, ^23^ and the time of onset of a full immune‐mediated response may take several weeks to develop,^24^, ^25^ early changes in pembrolizumab clearance were evaluated to assess the prognostic value of early changes in clearance. A 6‐week period was selected as the early‐change timeframe, as this period includes at least two pembrolizumab concentration measurements to inform the change of pembrolizumab clearance as this coincides with the first disease response evaluation conform RECIST 1.1 at 6 weeks.^17^

#### 2.2.2 Evaluation of the prognostic and predictive performance

The optimal cut points of baseline pembrolizumab clearance and the change in pembrolizumab clearance over the first 6 weeks of treatment were calculated using maximally selected rank statistics, as no established thresholds exist. The prognostic potential of early pembrolizumab clearance was evaluated by comparing PFS and OS differences between the two subgroups using log‐rank tests and Kaplan–Meier analysis. Additionally, a subgroup analysis was performed to assess the impact of chemotherapy addition on PFS and OS in patients with PD‐L1 ≥ 50% and either high pembrolizumab clearance at the first dose or a low decrease in clearance over the first 6 weeks of treatment.

Furthermore, sensitivity, specificity, positive predictive value (PPV) and negative predictive value (NPV) of the early pembrolizumab clearance as a prognostic biomarker for non‐response were calculated via standardized formulas,^26^ with disease control (progression‐free) at 6 months as the response definition, based on previous studies.

#### 2.2.3 Assessment of a limited sampling approach

The association of treatment non‐response with early clearance was assessed with clearance estimates from a rich pharmacokinetic dataset, with frequent pharmacokinetic sampling over a long time period. Since the purpose of our study was to assess clearance as an early prognostic biomarker, we quantified the predictive performance of a limited sampling strategy to estimate clearance based on a peak and trough sample after the first pembrolizumab dose and compared this with the predictive performance to quantify clearance in our dataset. We performed a Monte Carlo simulation of a limited sampling strategy (a peak and trough sample for each individual on days 1 and 21) in virtual patients receiving a 200 mg dose every 21 days. A second Monte Carlo simulation was performed with multiple replicates of the dataset and population for our pharmacokinetic study. Using the developed final pharmacokinetic model, we obtained the empirical Bayes estimates for lean body weight‐corrected clearance for both the limited sampling dataset and the dataset replicates of our study. Since the “true” simulated clearance values in these datasets were known, we could assess the accuracy of limited sampling against comprehensive pharmacokinetic sampling by calculating the MPE and evaluating its precision through the NMRSE.^27^

## 3 RESULTS

### 3.1 Population characteristics

Between October 2020 and May 2023, 303 patients with a median follow‐up of 12.9 months (range 0.7–67.1) were included in the analysis. Most patients had an ECOG PS of 0 or 1 (93%), PD‐L1 expression of <50% (60%), and received a combination of pembrolizumab and chemotherapy (74%). The population characteristics are shown in Table 1.

**TABLE 1:** Characteristics of the population (*N* = 303).

	Categorical variables *N* (%)	Continuous variables median (min–max)
Baseline characteristics		
Gender		
Female	135 (45)	
Male	168 (55)	
Age (years)	303 (100)	68 [31–93.8]
Bodyweight (kg)	303 (100)	72 [38–124.5]
Height (cm)	303 (100)	172 [148–203]
Albumin (g/L)	202 (67)	38.2 [21–50]
eGFR (mL/min/1.73m^2^)	156 (52)	90 [30–113]
ECOG PS		
0	102 (34)	
1	179 (59)	
2	19 (6)	
3	3 (1)	
PD‐L1 expression		
≥50%	121 (40)	
0–49%	182 (60)	
Smoking		
Current	112 (37)	
Former	182 (60)	
Never	9 (3)	
Packyears (years)	253 (84)	36 [1–143]
Treatment regimen		
Monotherapy	78 (26)	
Pembro‐pem‐plat	180 (59)	
Pembro‐pacli‐plat	45 (15)	
Outcomes		
Progression‐free survival (months)	303 (100)	9.6 [0.1–51.7]
Status progression‐free survival^a^		
Did not progress	73 (24)	
Progressed	198 (65)	
Lost to follow‐up/unknown status^b^	32 (11)	
Overall survival (months)	303 (100)	18.1 [0.7–58.8]
Status overall survival^a^		
Alive	122 (40)	
Dead	181 (60)	
Response		
Non‐responders	111 (37)	
Responders	192 (63)	
Plasma samples eligible for pharmacokinetic evaluation	1267 (100)	3 [1–16]

Abbreviations: ECOG PS, Eastern Cooperative Oncology Group Performance Status; eGFR, estimated glomerular filtration rate; PD‐L1, programmed cell death ligand 1; Pembro‐pacli‐plat, pembrolizumab‐paclitaxel‐platinum; Pembro‐pem‐plat, pembrolizumab‐pemetrexed‐platinum.

^a^ At data cut‐off (June 2024).

^b^ Patients without disease progression on the latest CT scan who died from unknown causes.

### 3.2 Early clearance as a prognostic biomarker for survival

#### 3.2.1 Pembrolizumab clearance outcomes

**FIGURE 1:**
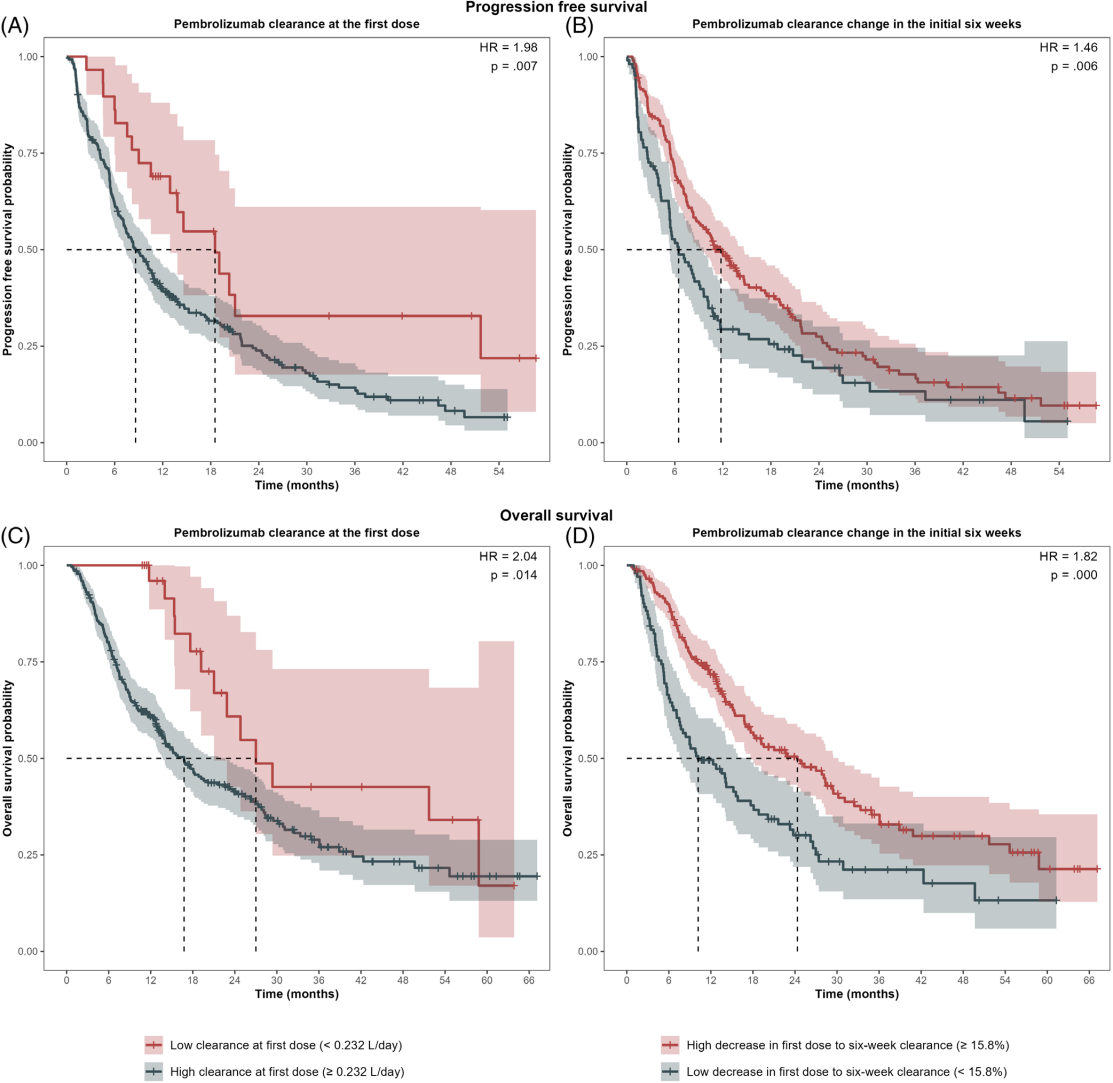
Kaplan–Meier curves displaying progression‐free survival in relation to pembrolizumab clearance at the first dose (A) and change in clearance between first dose and 6 weeks (B), as well as overall survival by clearance at the first dose (C) and change in clearance over 6 weeks (D).

The median lean body weight normalized pembrolizumab clearance at the first dose was 0.279 L/day. From the first dose to 6 weeks, the clearance decreased by a median of 21.7%. The cut points for clearance at the first dose (0.232 L/day) and change of pembrolizumab clearance between the first dose and 6 weeks (15.8% decrease), determined using maximally rank statistics, were identical for predicting PFS and OS. Patients with high lean body weight normalized pembrolizumab clearance at the first dose (≥ the cut point of 0.232 L/day) were statistically significantly more likely to have progressive disease compared to those with low clearance at the first dose (< the cut point of 0.232 L/day) (HR = 1.98, 95% CI [1.21, 3.26], *p* = .007) (Figure 1A). Moreover, patients who exhibited a low decrease to no decrease (< the cut point of a 15.8% decrease) in pembrolizumab clearance from first dose to 6 weeks were statistically significantly more likely to experience disease progression during the study period than those with high decrease (≥ the cut point of a 15.8% decrease) in pembrolizumab clearance (HR = 1.46, 95% CI [1.12, 1.92], *p* = .006) (Figure 1B). Similarly, both pembrolizumab clearance at the first dose and changes in pembrolizumab clearances over the first 6 weeks were significantly associated with death (HR = 2.04, 95% CI [1.16, 3.59], *p* = .014 and HR = 1.82, 95% CI [1.35, 2.45], *p* = .000, respectively) (Figure 1C,D).

The subgroup analysis on the addition of chemotherapy revealed no differences in PFS or OS (*p*‐value .261 to .935) by the addition of chemotherapy in PD‐L1 ≥ 50% and high pembrolizumab clearance at the first dose or low decrease in clearance over the first 6 weeks of treatment (Figure S3).

#### 3.2.2 Prognostic and predictive performance of early clearance

The performance of early lean body weight normalized pembrolizumab clearance to identify non‐response is presented in Table 2. Pembrolizumab clearance at the first dose distinguished non‐responders with a sensitivity of 0.96 and PPV of 0.39, indicating that among patients classified as non‐responders, 39% were actual non‐responders, experiencing disease progression within the first 6 months of treatment. In contrast, changes in pembrolizumab clearance over the first 6 weeks better distinguished responders, with a specificity of 0.72 and a NPV of 0.69, indicating that among patients classified as responders based on this biomarker, 69% were actual responders who did not experience disease progression within the first 6 months of treatment.

**TABLE 2:** Sensitivity and specificity of pembrolizumab clearance as a biomarker for non‐response.

	Sensitivity^a^ [95% CI]	Specificity^b^ [95% CI]	Positive predictive value	Negative predictive value
Clearance at first dose	0.96 [0.93, 1.00]	0.13 [0.08, 0.18]	0.39	0.86
Change in clearance in the initial 6 weeks	0.44 [0.35, 0.53]	0.72 [0.66, 0.79]	0.48	0.69

^a^ High sensitivity refers to a high proportion of true positive results. A positive test outcome indicates either a high clearance at the first dose or a minimal change in clearance. Additionally, a positive result signifies non‐response, defined as progression within a 6‐month timeframe.

^b^ High specificity refers to a high proportion of true negatives. A negative test outcome indicates either a low clearance at the first dose or a high change in clearance. Additionally, a negative result signifies response, defined as no progression within a 6‐month timeframe.

#### 3.2.3 Performance of a limited sampling approach

The predictive performance of a limited sampling strategy to estimate clearance resulted in an MPE of +4.5% and an NRMSE of 16.8%, which was similar to the respective values obtained from the pharmacokinetic sampling as performed in our study with an MPE of +3.2% and an NRMSE of 14.2%, showing that assessment of early clearance of pembrolizumab can be based on two samples.

## 4 DISCUSSION

The present study reveals that lean body weight normalized clearance of pembrolizumab is a highly sensitive biomarker for the identification of non‐responders to immune checkpoint inhibition in NSCLC patients. Both baseline clearance and early change in clearance can predict progression and death. Furthermore, we predict that based on two samples in the first 3 weeks of treatment, baseline clearance of pembrolizumab can be accurately and precisely assessed, offering a practical and reliable method to identify non‐responders early in treatment and timely inform therapeutic decisions.

Variations in pembrolizumab clearance are believed to result from both inter‐ and intraindividual differences in catabolism, the primary route of ICI metabolism and elimination, which occur in response to changes in disease burden following ICI‐containing treatment.^12^, ^23^, ^28^ Consequently, lower ICI plasma concentrations reflect non‐response to treatment, and increasing the dosage is unlikely to enhance therapeutic outcomes. This finding is underlined by the fact that no dose–response relationship was observed in the range of 2 mg/kg every 21 days to 10 mg/kg every 14 days in NSCLC patients.^29^

In agreement with this study, our previous research on the prognostic value of nivolumab clearance demonstrated that clearance at the first dose and early changes in clearance are prognostic for OS.^9^ The median clearance of 0.266 L/day in the nivolumab study was comparable to our findings of 0.279 L/day, suggesting that clearance mechanisms are likely similar. However, direct comparisons with our results for PFS or sensitivity and specificity are not possible, as the earlier study did not evaluate PFS and researched a population in exceptionally good condition. Consequently, a good prognosis is reflected in the high response rate. Our results also align with those of Maritaz et al., who reported that low nivolumab clearance or high nivolumab plasma concentrations in early cycles correlate with improved survival.^30^ However, their study compared groups based on the median values rather than an optimally discriminative cut point derived from statistical methods such as maximally selected rank statistics or ROC analysis. Without an optimal cut point, the discriminative effect may have been underestimated. Additionally, a Japanese study investigating the prognostic value of pembrolizumab reported comparable findings but estimated a median clearance of approximately half that of ours at 0.104 L/day. This discrepancy cannot be attributed to differences in body size between Japanese and Western populations, as both studies adjusted for body size, using body surface area in the Japanese study and lean body weight in ours. Notably, the Japanese study also concluded that their observed clearance was approximately half of the range historically reported in Western populations (range 0.22–0.257 L/day).^23^

Our study had some limitations. For clinical use, particularly in rapidly progressing diseases like NSCLC, high PPV is crucial to ensure sufficient certainty before discontinuing treatment in patients classified as non‐responders. A PPV threshold of at least 0.95 would mean that 95% of patients classified as non‐responders are true non‐responders (i.e., those who experience disease progression within the first 6 months of treatment), thereby minimizing the risk of incorrectly stopping effective treatment. In our analysis, the PPV did not exceed 0.48. Consequently, the PPV of this biomarker should be increased substantially to facilitate clinical implementation, potentially limiting the immediate clinical impact of our findings. This limitation in predictive power could be addressed by identifying the optimal pembrolizumab clearance cut point that maximizes the PPV and integrating early pembrolizumab clearance with other biomarkers. Such a panel of biomarkers could enhance the personalization of treatment follow‐up, allowing for less intensive monitoring.

Another potential limitation for clinical implementation is that pembrolizumab serum concentrations require quantification using an enzyme‐linked immunosorbent assay (ELISA). Although this technique is routinely employed in clinical laboratories, with a short turnaround time and relatively low costs (approximately 30 to 40 Euros per sample), access may be limited in lower‐income countries due to the restricted availability of the necessary assays.^31^ Despite some limitations, this study has several notable strengths. The dataset represents a large representative real‐world population of patients with NSCLC receiving pembrolizumab‐containing treatment, featuring extensive sampling of pembrolizumab plasma concentrations (1267 samples from 303 patients) and survival data, which enables accurate estimation of the prognostic value of pembrolizumab clearance. Treatment response was assessed frequently, and the follow‐up period was extensive, with a median 12.9 months (range 0.7–67.1). Most patients experienced a survival event (PFS 65% and OS 60%), allowing for reliable analyses of PFS and OS. Furthermore, clearance of the exogenous pembrolizumab offers an advantage in biomarker applications due to the known dosing and sampling details (dose, administration date and time, sampling date and time), allowing for more precise clearance estimation compared to endogenous substances like albumin. Notably, we demonstrated that clearance can be reliably assessed using only two blood samples within the first 3 weeks of treatment, which enhances the clinical feasibility of clearance as a prognostic biomarker.

Our results illustrate that early pembrolizumab clearance is a highly sensitive prognostic biomarker for survival. It is also a clinically feasible biomarker, as it can be assessed using only two samples: one directly after the first dose and one at the trough. However, its PPV might have to be improved. These findings hold significant relevancy in the current era, where responses to pembrolizumab therapy exhibit considerable variability, necessitating a clear differentiation between responders and non‐responders.

## AUTHOR CONTRIBUTIONS

**Fenna de Vries:** Conceptualization; methodology; investigation; validation; formal analysis; data curation; visualization; writing – original draft; writing – review and editing. **Leila‐Sophie Otten:** Conceptualization; methodology; data curation; investigation; validation; formal analysis; visualization; writing – original draft; writing – review and editing. **Berber Piet:** Conceptualization; writing – original draft; writing – review and editing. **Eric J. F. Franssen:** Conceptualization; writing – original draft; writing – review and editing; supervision. **Arthur A. J. Smit:** Conceptualization; writing – original draft; writing – review and editing. **Michel M. van den Heuvel:** Conceptualization; writing – original draft; writing – review and editing. **Rob ter Heine:** Conceptualization; methodology; writing – original draft; supervision; writing – review and editing.

## FUNDING INFORMATION

The DEDICATION‐1 study was funded by Stichting Treatmeds, a public organization financially supported by Dutch Health Insurers. The PD‐1 study was funded by transformation funds of the Dutch Health Insurers.

## CONFLICT OF INTEREST STATEMENT

Rob ter Heine received research funding from Amgen and Stichting Treatmeds, as well as fees for acting as an advisory board member for Samsung Bioepis. All other authors have no competing interests.

## ETHICS STATEMENT

The DEDICATION‐1 study (ClinicalTrials.gov ID NCT04909684) was approved by METC‐Oost Nederland under the WMO. The PD‐1 study, exempt from WMO, was approved by MEC‐U (W20.230) and the OLVG institutional review board, with consent secured accordingly. Informed consent for participation and publication was obtained from patients.

## Supporting information


**DATA S1.**Supporting information.

## Data Availability

The data that supports the findings of this study are available in the supplementary material of this article. Further information is available from the corresponding author upon request.
