# Supplementary material for: Early pembrolizumab clearance as prognostic biomarker for non‐response in patients with advanced non‐small cell lung cancer
Source: Int J Cancer. 2025 Jul 24;157(12):2569–76. doi: 10.1002/ijc.70052 (PMC12541564; doi:10.1002/ijc.70052)
Supplement: Supplementary file 1 — DATA S1.Supporting information. [file IJC-157-2569-s001.pdf]

Early Pembrolizumab Clearance as Prognostic Biomarker for Non-Response in Patients with Advanced Non-Small Cell Lung Cancer

AUTHORS

Fenna de Vries, Leila-Sophie. Otten, Berber. Piet, Eric J.F. Franssen, Arthur A.J. Smit, Michel M. van den Heuvel, Rob ter Heine, The DEDICATION-1 Study group

TABLE OF CONTENTS

Supplementary Materials & Methods **2**
Supplementary Materials & Methods 1. Bioanalytical methods and their comparison 2
Supplementary Materials & Methods 2. Population pharmacokinetic model 4

Supplementary Tables **8**
Supplementary Table 1. Parameter estimates of the final pharmacokinetic model 8

Supplementary Figures **9**
Supplementary Figure 1. Comparison of the outcomes of analytical methods 9
Supplementary Figure 2.1. Schematic overview of the pharmacokinetic model 10
Supplementary Figure 2.2. Goodness-of-fit plots 11
Supplementary Figure 2.3. Prediction-corrected visual predictive checks plots 12
Supplementary Figure 3. Kaplan Meier curves of patients with PD-L1 ≥ 50% 13

Supplementary References **14**

Supplementary Materials & Methods

Supplementary Materials & Methods 1. Bioanalytical methods and their comparison

General description of the bioanalytical methods

For samples collected from patients in the PD-1 study, an ELISA was performed by Sanquin Diagnostic Services (Amsterdam, the Netherlands), which was validated following the U.S. Food and Drug Administration (FDA) guidelines. This method had a mean accuracy of 97 to 105% from the lower limit of quantification of 0.1 µg/mL to the upper limit of quantification of 200 µg/mL and coefficients of variation between 3.0 and 9.9%. For samples from patients in the DEDICATION-1 study, a ultra-performance liquid chromatography-tandem mass spectrometry (UPLC-MS/MS) method was performed by Radboudumc, which was validated per the European Medicines Agency (EMA) and FDA guidelines. [1, 2] This method had a lower limit of quantification of 2.5 µg/mL and a limit of detection of 1.25 µg/mL. A method comparison was performed to align sample concentrations measured by the different methods. The pembrolizumab concentrations of samples from the PD-1 study were converted based on the outcomes of this method comparison.

Comparison of the bioanalytical methods

This study compared the two methods for quantifying pembrolizumab levels in plasma to evaluate their agreement and the feasibility of combining their results. Of all samples initially quantified by ELISA, 45 were re-analyzed using the UPLC-MS/MS method, successfully analyzing 43 samples. The ELISA-quantified pembrolizumab concentrations of these samples ranged from 2.51 to 157.00 mg/L, with a median of 22.7 mg/L.

A linear regression analysis was performed to assess the relationship between the two methods. Pearson's correlation coefficient was calculated to measure the strength and direction of the relationship. The intercept and slope of the best-fit regression line were examined to identify biases. A Bland-Altman plot was constructed to identify any systematic bias and visualize the range within which most differences lie. The mean difference and the limits of agreement (mean ± 1.96 standard deviations) were calculated. The regression equation obtained was:

$$(1)\ \ ELISA = -2.07 + 1.20 * UPLC - MS/MS$$

This equation along with a Pearson's correlation coefficient of 0.961, indicate a strong positive correlation (**Supplementary Figure 1A**). The 95% confidence intervals for the slope and intercept were 1.093 to 1.310 and -7.708 to 3.559 respectively (**Supplementary Figure 1B**), suggesting a significant linear relationship with minor systematic and proportional biases.

The Bland-Altman plot (**Supplementary Figure 1C**) displays a mean difference between the UPLC-MS/MS and ELISA methods of 5.99 mg/L. The limits of agreement ranged from -20.78 to 32.76 mg/L,

indicating that most differences between the methods' outcomes lie within these limits, showing a generally acceptable level of agreement.

These analyses demonstrate a strong correlation and reasonable agreement between the UPLC-MS/MS and ELISA methods for quantifying pembrolizumab in plasma, suggesting the feasibility of combining results after correction for differences using the best-fit equation:

(2) $$Pembrolizumab\ concentration_{ELISA-converted} = \frac{Pembrolizumab\ concentration_{ELISA} + 2.07}{1.20}$$

Supplementary Materials & Methods 2. Population pharmacokinetic model

Methods

Non-linear mixed effects modelling (NONMEM) was performed using the software package NONMEM v7.51 (ICON, Dublin, Ireland), using the first order conditional estimation method with interaction (FOCE-I). Observed pembrolizumab data were fitted to a two-compartment pharmacokinetic (PK) model incorporating with linear first-order clearance (CL) from the central compartment. Further disposition was described with intercompartmental clearance (Q), central compartment volume of distribution (V_1) and peripheral compartment volume of distribution (V_2) (**Supplementary Figure 2.1**).

All model parameters were scaled allometrically to lean body weight (LBWT) to account for interindividual body weight variation, normalized to a reference LBWT of 52.5 kg. For reference, a LBWT of 52.5 kg corresponds to a typical individual weighing 70 kg, with a height of 170 cm and a body mass index (BMI) of 24 kg/m² in a population with an assumed 50:50 male-to-female ratio. LBWT was calculated using the sex-specific James equation [3]:

$$(1)\ \ LBWT_{Male} = (1.1 * BWT) - (0.0128 * BMI * BWT)$$

$$(2)\ \ LBWT_{Female} = (1.07 * BWT) - (0.0148 * BMI * BWT)$$

where body weight (BWT) is expressed in kilograms and BMI in kilograms per square meter (kg/m²). In cases of missing BWT data, two imputation strategies were applied. Carry-forward or backward imputation was used to calculate missing values before the first or after the last known measurements. Linear interpolation was used to calculate missing BWT values between two known measurements.

Pembrolizumab CL was modeled as the product of clearance at the first dose (CL_I) and a time-dependent component (TDPK) to capture the observed changes in drug elimination over time:

$$(3)\ \ CL = CL_I * TDPK$$

Clearance at the first dose was expressed as:

$$(4)\ \ CL_I = \theta_3 * \left(\frac{LBWT}{52.5}\right)^{\theta_1} * \exp^{\eta_1}$$

Where θ_3 is the typical value of CL (0.277 L/day), θ_1 is the allometric exponent for clearance (0.882), and η_1 represents the interindividual variability (IIV) in clearance, with an estimated variance of 0.037.

The time-dependent component was modeled using a sigmoidal relationship:

$$(5)\ \ TDPK = \exp\left(\frac{I_{max} * TIME^{HILL}}{{TI_{50}}^{HILL} + TIME^{HILL}}\right)$$

where I_{max} represents the maximum fractional change in clearance from its initial value, TI_{50} is the time (in days) required to reach half of the maximum change, and the HILL is the Hill coefficient describing the steepness of the time-dependency. The estimated values for these parameters were -0.562 for I_{max}, 48.8 days for TI_{50}, and 1.62 for the Hill coefficient (**Supplementary Table 1**). IIV was also estimated for TI_{50} with an estimated variance of 2.06. Time-dependent clearance was assessed using ordinary differential equation solver (ADVAN13) with time as a constant factor. For model stability and reduced run times and because negligible differences in estimations, we switched to ADVAN3.

Volumes of distribution (V_1 and V_2) and Q were similarly allometrically scaled and included IIV, except for Q. The general parametrization for these variables was:

$$(6)\ P = \theta_{x1} * \left(\frac{LBWT}{52.5}\right)^{\theta_{x2}} * \exp^{\eta_x}$$

where P represents the parameter of interest, θ_{x1} is its typical value, θ_{x2} denotes the estimated allometric scaling exponent (0.807 for V_1 and V_2, and 0.882 for Q and CL), and η_x accounts for the IIV of the parameter. The typical values for V_1, Q, and V_2 were estimated at 3.35, 1.06, and 4.45, respectively, and the IIV for V_1 and V_2 were 0.018 and 0.134, respectively (Supplementary Table S1).

The individually predicted pembrolizumab concentrations were calculated as follows:

$$(7)\ IPRED = \frac{A(1)}{V_1}$$

Where A(1) represents the amount measured in the central compartment and V_1 the central volume of distribution. Residual variability on observed pembrolizumab concentrations was described with a proportional error model:

$$(8)\ Y = IPRED + (IPRED * ERR_1)$$

where Y represents the observed pembrolizumab concentration, IPRED is the individual predicted concentration, and ERR_1 represents the proportional error term. Model improvements were evaluated through assessments of parameter precision, reductions in the objective function value, shrinkage, conditional number, and residual variability. Goodness-of-fit plots and visual predictive checks were inspected visually to evaluate model performance. The robustness of the parameter estimates was evaluated by performing a sampling importance resampling procedure. [4]

The goodness-of-fit plots show that the model's predictions align with the measured pembrolizumab concentrations, and that variability is symmetrically distributed over time (**Supplementary figure 2.2**). Furthermore, in the prediction-corrected visual predictive check plots (**Supplementary figure 2.3**), the alignment of observed data with the median prediction and prediction intervals is consistent across

most of the time-after-dose profile, particularly during the early phases, where data are more densely populated.

NONMEM Model code

```
$PROBLEM        PEMBROLIZUMAB real world PK
$INPUT          (...)
$DATA           (...)
$SUBROUTINE     ADVAN3 TRANS4

$PK
ALLOLEAN_CL = (LBWT/52.5) ** THETA(1)
ALLOLEAN_V  = (LBWT/52.5) ** THETA(2)

IMAX = THETA(7)
TI50 = THETA(8) * EXP(ETA(5))
HILL = THETA(9)

CLI = THETA(3) * ALLOLEAN_CL * EXP(ETA(1))
V1  = THETA(4) * ALLOLEAN_V * EXP(ETA(2))
Q   = THETA(5) * ALLOLEAN_CL * EXP(ETA(3))
V2  = THETA(6) * ALLOLEAN_V * EXP(ETA(4))

TDPK = EXP(IMAX * (TIME**HILL) / (TI50**HILL + TIME**HILL))
CLWT = THETA(3)* EXP(ETA(1)) * TDPK

CL = CLI  *  TDPK

S1 = V1

$ERROR
IPRED = A(1)  /   V1

Y = IPRED + (IPRED * ERR(1))

$THETA
0.882           ; 1 ALLOLEAN_CL
0.807           ; 2 ALLOLEAN_V
0.277           ; 3 CL
3.35            ; 4 V1
1.06            ; 5 Q
4.45            ; 6 V2
-0.562          ; 7 IMAX
48.8            ; 8 TI50
1.62            ; 9 HILL

$OMEGA
0.0366          ; 1 CL
0.0182          ; 2 V1
0 FIX           ; 3 Q
0.134           ; 4 V2
2.06            ; 5 T50

$SIGMA
0.0291           ; PROP ERR

$ESTIMATION METHOD=1 INTERACTION MAXEVAL=4000 NSIG=1 PRINT=1 NOHABORT
$COVARIANCE PRINT=E UNCONDITIONAL MATRIX=S
```

Supplementary Tables

Supplementary Table 1. Parameter estimates of the final pharmacokinetic model

Parameter	Estimates [95% CI]
**Population values**	
Allometric exponent for CL_I and Q	0.822 [0.716, 1.052]
Allometric exponent for volume parameters	0.807 [0.612, 1.011]
CL_I (L/day)	0.227 [0.257, 0.302]
V_1 (L)	3.355 [3.198, 3.547]
Q (L/day)	1.065 [0.658, 1.826]
V_2 (L)	4.451 [3.923, 5.112]
I_{MAX}	-0.562 [-0.669, -0.456]
TI_{50} (days)	48.764 [36.388, 58.849]
HILL	1.625 [1.190, 2.064]
**Interindividual variability**	
CL_I (variance)	0.037 [0.027, 0.049]
V_1 (variance)	0.018 [0.008, 0.034]
V_2 (variance)	0.134 [0.079, 0.202]
TI_{50} (variance)	2.062 [1.263, 2.977]
**Residual error**	
Proportional error (variance)	0.029 [0.027, 0.032]

Note. CL_I = clearance at the first dose; HILL = hill coefficient; IIV = inter-individual variability; I_{MAX} = maximum change in clearance from its initial value; OBJV = objective function value; Q = intercompartmental clearance; TI_{50} = time to reach half the maximum inhibitory effect; V_1 = volume of central compartment; V_2 = volume of peripheral compartment.

Supplementary Figures

Supplementary Figure 1. Comparison of the outcomes of analytical methods

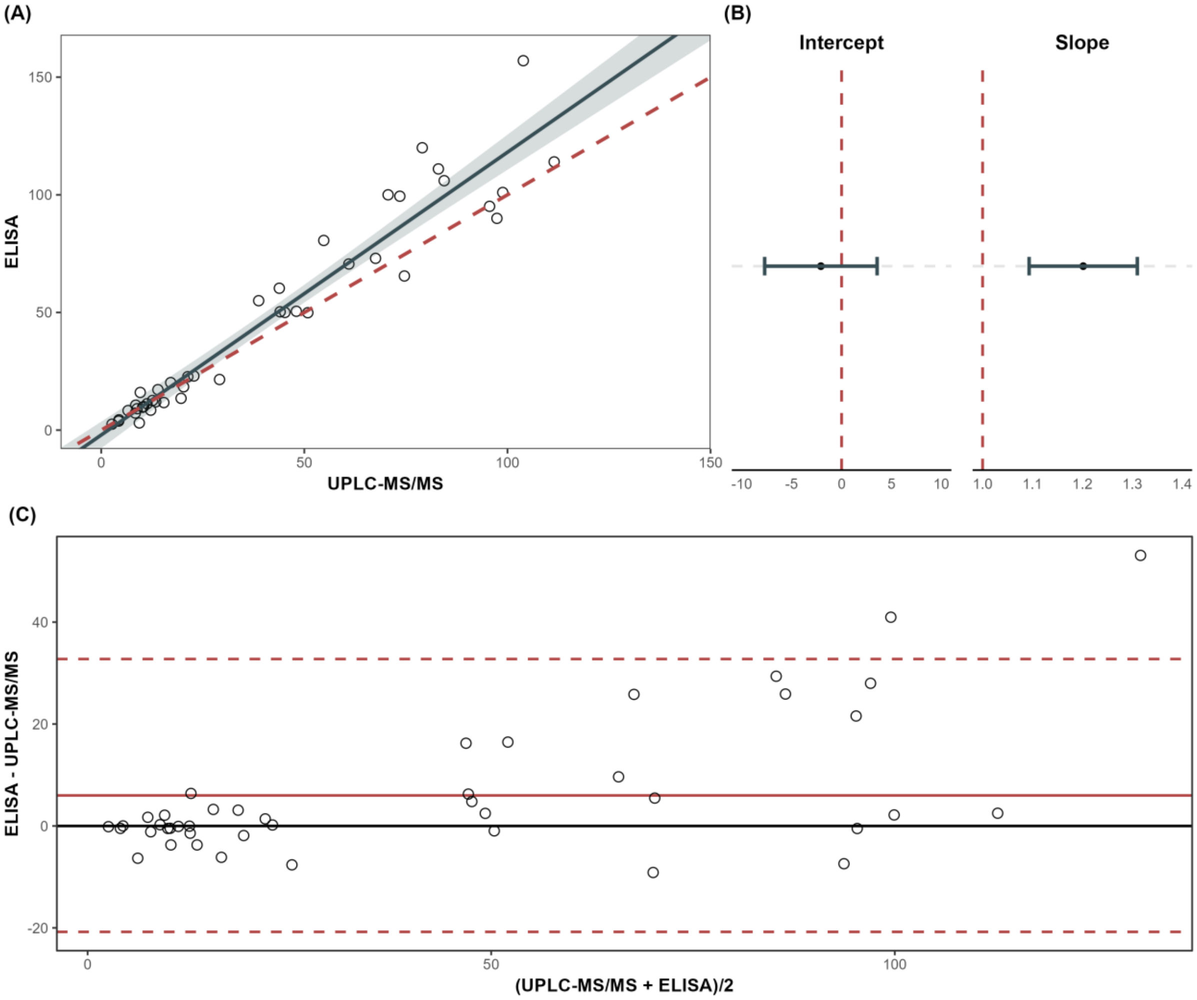

Supplementary figure 1: *Comparison of the outcomes of both analytical methods by means of linear regression (A), the 95% confidence intervals of the interval and slope (B) and the Bland-Altman plot (C).*

Supplementary Figure 2.1. Schematic overview of the pharmacokinetic model

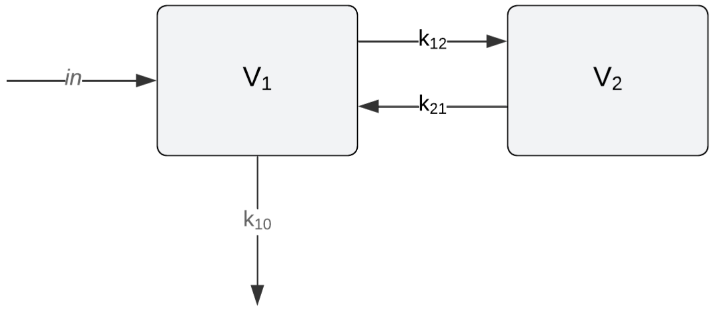

Supplementary figure 2.1. *Schematic overview of the pharmacokinetic model with linear first-order clearance following intravenous infusion. Compartment* V_1 *represents the central compartment and compartment* V_2 *the peripheral compartment. The model was parameterized with the constants* k_{10}*,* k_{12} *and* k_{21}*; with* k_{10} *representing the rate constant for elimination from the central compartment calculated by* $k_{10} = CL/V$*;* k_{12} *representing the intercompartmental distribution rate constant from the central to the peripheral compartment calculated by* $k_{12} = Q/V_1$*; and* k_{21} *representing the intercompartmental distribution rate constant from the peripheral to the central compartment calculated by* $k_{21} = Q/V_2$*.*

Supplementary Figure 2.2. Goodness-of-fit plots

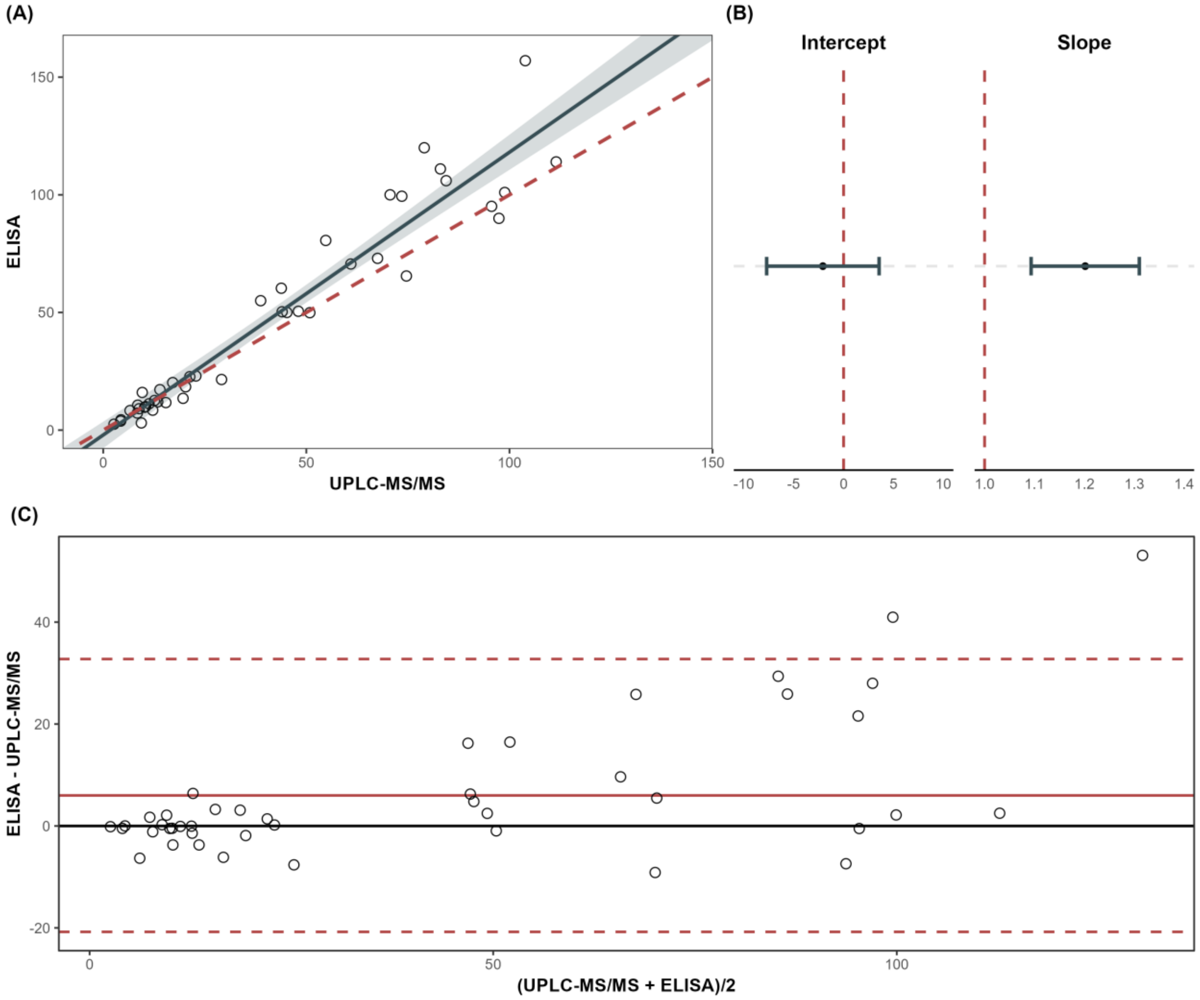

Supplementary Figure 2.2. *Goodness-of-fit plots of the PK model for pembrolizumab. Displayed in the top panels are the individual predicted concentrations versus observed concentrations of the model (A) and the population predicted concentrations versus observed concentrations of the mode (B) and displayed in the bottom panels are the population predicted concentrations versus the conditional weighted residuals (C) and the conditional weighted residuals versus the time in days (D).*

Supplementary Figure 2.3. Prediction-corrected visual predictive checks plots

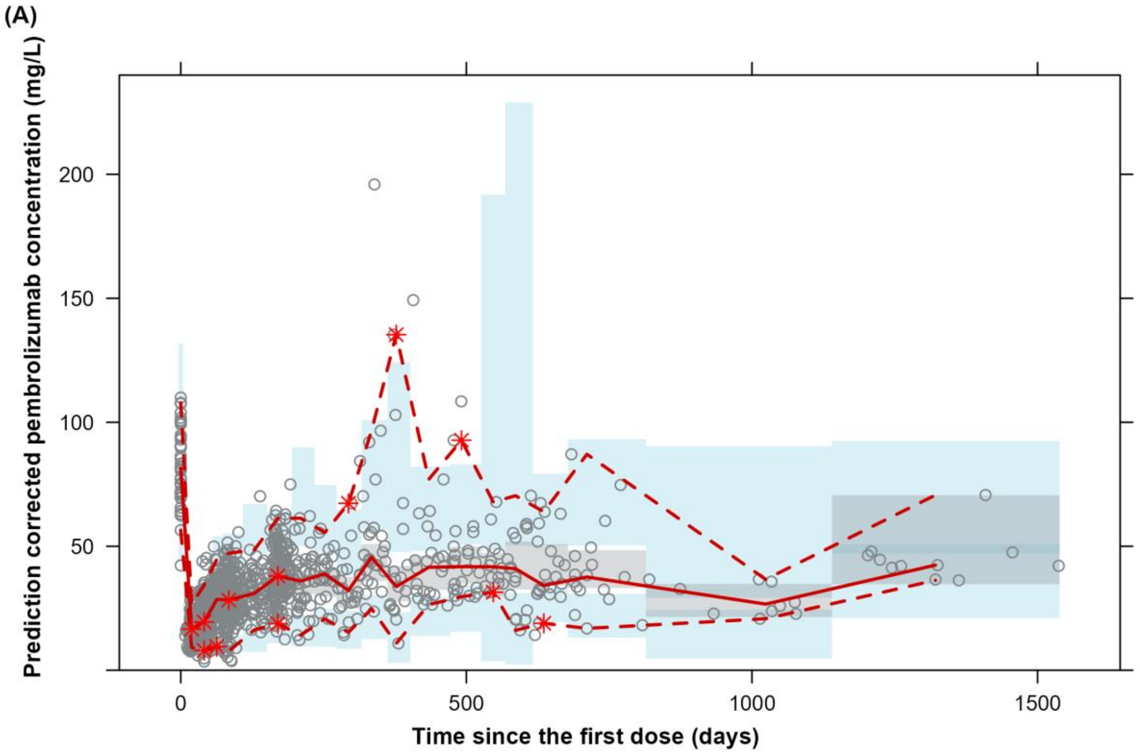

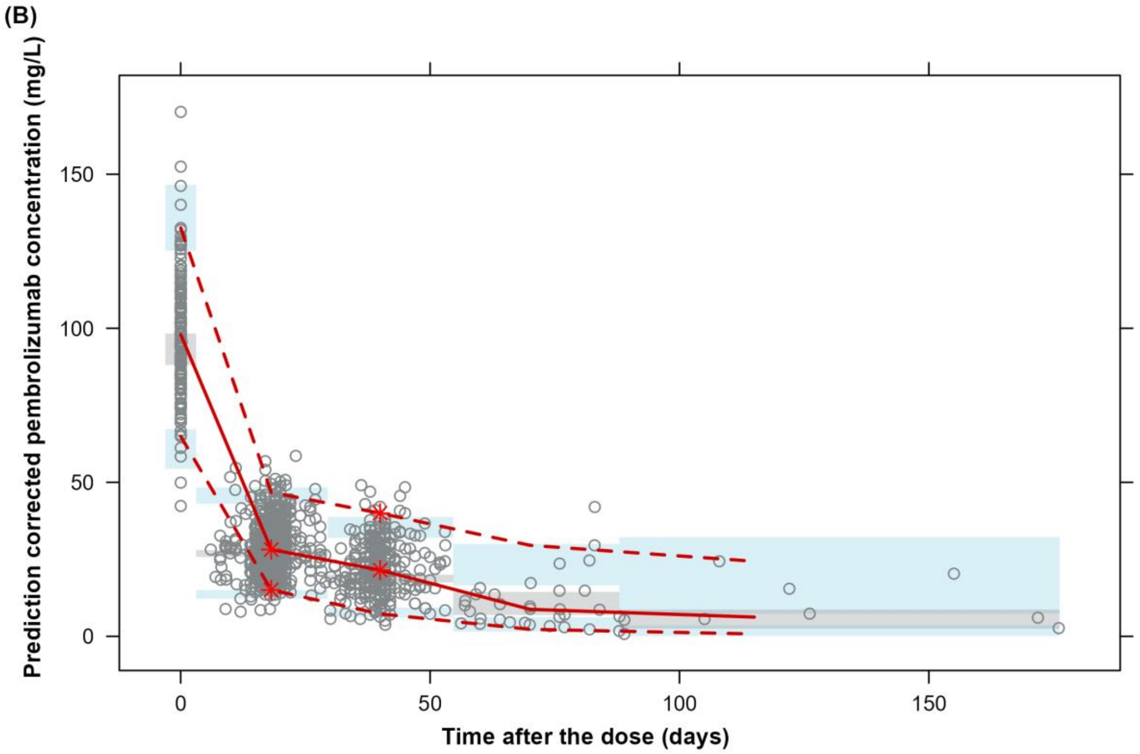

Supplementary Figure 2.3. *Prediction-corrected visual predictive checks plots. Showing the fit of predicted pembrolizumab concentrations versus the observed concentrations over time since treatment start (days) (A) and over time after dose (days) (B). The shaded blue areas represent the 95% prediction intervals for the simulated percentiles, while the red dashed lines indicate the 5th, 50th (median), and 95th percentiles of the simulated data. The observed concentrations (n = 200 samples) are overlaid as black open circles. The red asterisks (*) denote instances where the prediction-corrected observed pembrolizumab concentration at a specific time point falls outside the prediction intervals.*

Supplementary Figure 3. Kaplan Meier curves of patients with PD-L1 ≥ 50%

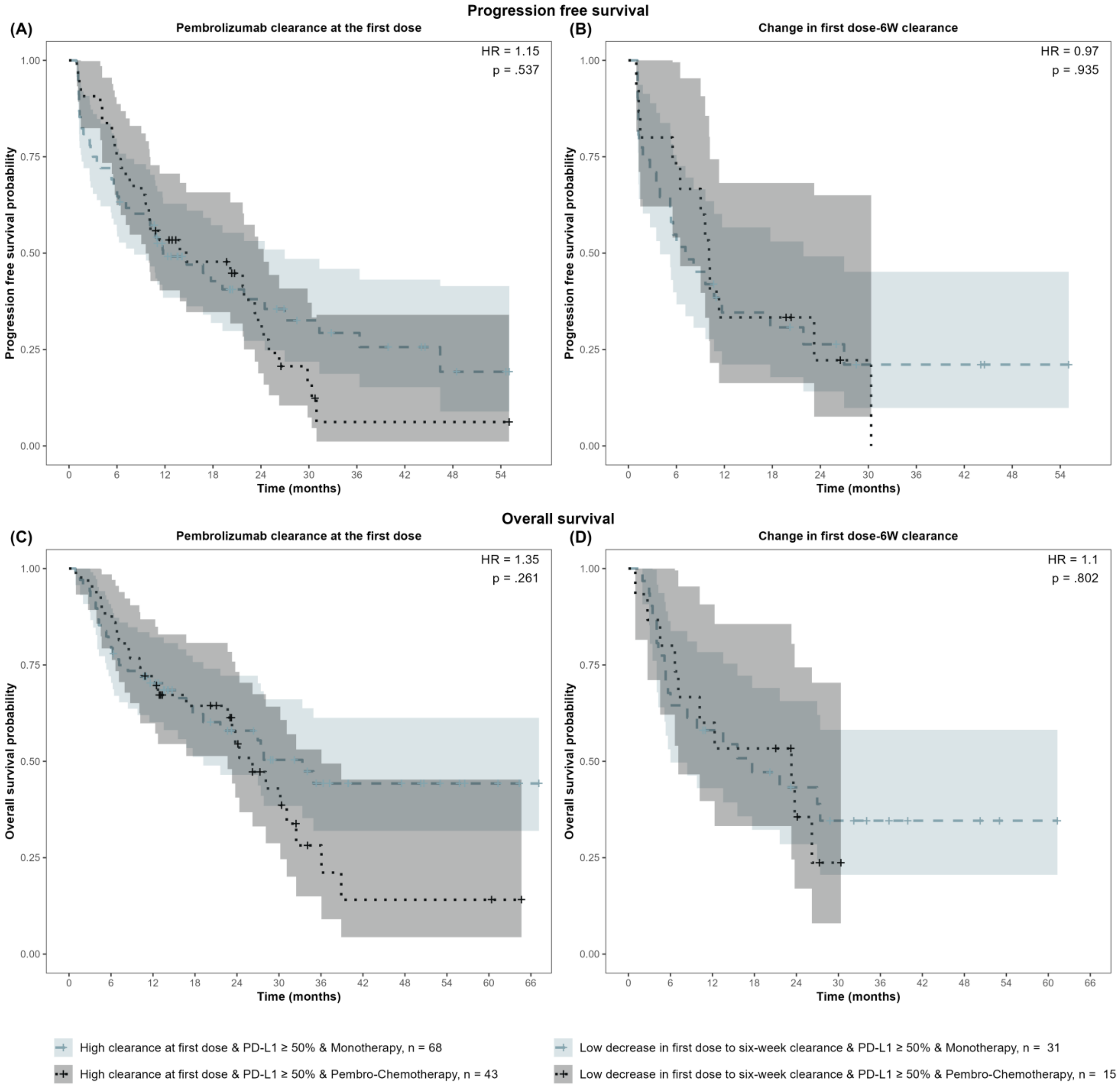

Supplementary Figure 3. *Kaplan-Meier curves with survival outcomes for patients with high PD-L1 status (≥ 50%) and a poor prognosis (defined by high baseline pembrolizumab clearance and a low decrease in clearance). Progression-free survival is shown by clearance at the first dose (A) and the change in clearance at six weeks (B). Overall survival is represented by clearance at baseline (C) and the change in clearance over the six-week period (D).*

Supplementary References

1. U.S. Food and Drug Administration. **Bioanalytical Method Validation Guidance for Industry**. 2018.
2. European Medicines Agency. **Guideline on Bioanalytical Method Validation**. 2011.
3. James WPT. **Research on obesity**. *Nutr Rev 1977,* **35**(9):249-52.
4. Dosne AG, Bergstrand M, Harling K, Karlsson MO. **Improving the estimation of parameter uncertainty distributions in nonlinear mixed effects models using sampling importance resampling**. *J Pharmacokinet Pharmacodyn 2016,* **43**(6):583-96.
